# An exploratory analysis of the interactions between social norms and the built environment on cycling for recreation and transport

**DOI:** 10.1186/s12889-018-6075-4

**Published:** 2018-10-05

**Authors:** Matthew Bourke, Toni A Hilland, Melinda Craike

**Affiliations:** 1 Institute for Health and Sport (IHES), Victoria University, PO Box 14428, Melbourne, VIC 8001 Australia; 2 School of Education, College of Design and Social Context, RMIT, PO Box 71, Melbourne, VIC 3083 Australia

**Keywords:** Interactions, Social norms, Built environment, Ecological models, Transport, Recreation, Cycling

## Abstract

**Background:**

There is growing evidence of the public health benefits of promoting cycling. The ways that the built environment and perceived social norms independently influence cycling participation is well established. However, whether these factors interact to influence cycling participation has not been examined. Such research is important because understanding the effect of multiple socio-ecological factors and the interactions between them is needed to guide the development of interventions and strategies to increase cycling participation. Therefore, the aim of this study is to explore the interactive effects of the built environment and perceived social norms on transport and recreational cycling.

**Methods:**

Data was collected using a self-administered online questionnaire from 228 office workers in Metropolitan Melbourne, Australia. Measures used in previous research were employed to assess self-reported transport and recreation cycling in the last week, perceptions of neighbourhood built environment, perceived social norms towards cycling, and objective land-use mix, residential density and street connectivity of the suburbs in which participants lived and work. Multiple binary logistic regression analyses were conducted to explore the interactive effects of the built environment and perceived social norms on transport and recreation cycling. All interactive effects were considered significant at *p* < 0.10.

**Results:**

There was a significant interactive effect between the workplace built environment and perceived group norm on transport cycling (*p* = 0.06). There were no other significant interactive effects observed between components of the built environment and perceived social norms on transport or recreational cycling.

**Conclusions:**

The interactive effect found in this study provides some evidence that the workplace built environment interacts with perceived group norms to influence cycling for transport. Positive perceptions of the workplace built environment, such as showers and secure bike racks, can somewhat compensate for the negative influence of when cycling is considered less of a norm among, family, friend or colleagues. However, the findings of this study did not support that the neighbourhood built environment and perceived social norms interact to influence cycling for recreation or transport. These findings contribute to the knowledge of how multiple factors may reciprocate to influence individual’s decision to cycle. More research into the interactive effects of socio-ecological factors is warranted.

**Electronic supplementary material:**

The online version of this article (10.1186/s12889-018-6075-4) contains supplementary material, which is available to authorized users.

## Background

There is growing evidence demonstrating the public health benefits of promoting participation in cycling [1–3]. This is because cycling for recreation and transport are positively related to overall physical activity [4–8], and improving participation in cycling has the potential to significantly increase the proportion of the population that accumulate health enhancing levels of physical activity [9, 10]. However, available data shows that less than 3% of total trips made by adults are by bicycle in the United States, Australia, Canada, Ireland, and United Kingdom, and less than 6% in France, Austria, and Switzerland [11]. Accordingly, the World Health Organisation suggests that more evidenced-based strategies and interventions to stimulate cycling participation are required [12]. To guide the development of such strategies and interventions it is important to understand the effect of multiple socio-ecological factors and the interactions that may exist between these factors [13].

Socio-ecological models recognise that physical activity is a complex and multi-faceted behaviour that may be influenced by multiple factors from various levels of influence [13, 14]. Socio-ecological models also suggest that synergistic relationships may exist between factors from different levels of influence [15, 16], and that a change at one level of influence is likely to have a reciprocal effect on other levels [17]. Therefore, socio-ecological models posits the effect of factors from multiple levels of influence on behaviour is likely to be greater than the summation of each individual factor [15]. Factors from the ecological model that may influence cycling include the built environment and social norms, which are the normal and accepted behaviours within a group of individuals [18].

There has been some research, albeit limited, reporting the interactive effects of the built environment and social environment on general recreational physical activity, walking for recreation and walking for transport in adults [19–23]. Two studies reported a synergistic interaction between the built environment and social support, where a supportive built environment and positive social climate reinforce each other to facilitate recreational walking [20], and transport walking [21]. In contrast, two studies suggest that the built and social environments interact in a way where a supportive built environment can compensate for a negative social environment for general recreational physical activity [22] and recreational walking [19]. Additionally, one study found no interaction between social norms or social support and walkability on adults transport or recreational walking [23].

Despite the independent influence of social norms and the built environment on cycling that has been reported previously [24–26], to the authors’ knowledge, the interactive effect of the built environment and social norms on cycling participation in adults is yet to be reported. To our knowledge, the only studies to investigate the interactive effects of the built and social environment on cycling have been conducted on children and adolescents between the ages of 9 and 16 [27, 28]. A study involving Belgian children reported an interactive effect between support from friends and neighbourhood walkability on cycling in leisure time [27]. The study showed that friend support moderated the relationship between walkability and cycling in leisure time; however the effect size of this interaction was small. The same study showed that there was no interactive effect between social norms, or support from parents and neighbourhood walkability on cycling in leisure time. Additionally, two studies found that there was no interactive effect between perceived social support and the built environment on children’s and adolescents’ active commuting behaviours [27, 28].

Inconsistent findings between different age groups and types of physical activity suggests the interactive effects of the built and social environments are likely to be behaviourally and contextually specific [29–31]. Therefore, further research is needed into specific physical activity behaviours, such as cycling for recreation and transport. The aim of the present study is to explore the interactive effects of the built environment and perceived social norms on adults’ participation in cycling for recreation and transport.

## Methods

### Study design

Cross-sectional data was collected between July and August 2017 using a self-administered online questionnaire (Qualtrics; see Additional file 1). Participants included 228 adult office workers (53% females) aged between 22 and 70 years (M = 38.92, SD = 10.85) from Metropolitan Melbourne, Australia. Participants were recruited from Metropolitan Melbourne due to the availability of geographic information system data and because there is a variability in cycling environments between neighbourhoods [32].

To recruit participants, an email introducing the study with a hyperlink to the questionnaire was sent to a convenience sample of organisations and workplace bicycle user groups in Metropolitan Melbourne. Bicycle user groups were identified from a publically accessible database [33], and researchers directly contacted several large organisations known to them. Organisations that chose to be involved in the study were prompted to distribute the questionnaire hyperlink to employees using internal communication channels. Due to the nature of the recruitment process, response rates could not be calculated. However, several strategies were used to increase the response rate of both cyclists and non-cyclists; namely the questionnaire length was kept short (i.e. less than 10 min to complete), which was highlighted in the recruitment email [34], and the study introductory email emphasised that employees were eligible to complete the questionnaire regardless of whether they cycle or not. Additionally, to encourage participation in this study, participants who completed the questionnaire were eligible to go into the draw to win one of five $50 department store gift cards.

Prior to the main study, the questionnaire was piloted by a convenience sample of six adults with similar characteristics of the study population. Based on their feedback slight modifications were made to the wording of some questions to meet Australian vernacular, and the layout of some questions was changed to improve questionnaire flow.

### Measures

#### Socio-demographic characteristics

Socio-demographic variables measured were gender, age, education level, number of cars in participant’s household, whether the participant had regular access to a bicycle, distance participants lived from their workplace, suburb of residency, and suburb of workplace.

#### Cycling participation

*Cycling for transport* was measured using an item from the International Physical Activity Questionnaire Long Form (IPAQ-LF) self-administered format [35]. The IPAQ-LF has good test-retest reliability [35], and has been used widely to measure cycling for transport [36, 37]. Participants were asked “during the last 7 days, on how many days did you cycle for at least 10 minutes to go from place to place?” Participants were also asked “how many minutes did you usually spend on one of these days to cycle from place to place?” Participants were instructed to only include cycling to get from place-to-place such as work, shops, and public transport. This wording was slightly modified from the original IPAQ-LF to more accurately reflect the Australian cycling context. In the present study, the distribution of time cycling for transport in the last week was skewed (46% of participants had not cycled for transport in the last week), and a decision was made to dichotomise the variable. The dichotomous categories were “cycled for transport in the last week” and “did not cycle for transport in the last week”.

*Cycling for recreation* was measured using an item derived from the IPAQ-LF [35]. Similar to a previous study [38], the question was adapted to only measure cycling for recreation. Participants were asked “during the last 7 days, on how many days did you bicycle for at least 10 minutes in your leisure time?” Participants were also asked “how many minutes did you usually spend on one of these days bicycling for recreation, sport, exercise, or leisure?” Participants were instructed to only include cycling that was solely for sport, exercise or leisure, and not to include any cycling that they had already reported. The distribution of time cycling for recreation in the last week was skewed (75% of participants had not cycled for recreation in the last week), so it was decided to dichotomise the variable. The dichotomous categories were “cycled for recreation in the last week” and “did not cycle for recreation in the last week”.

#### Perceived neighbourhood built environment

Perceptions of the built environment were measured using nine items from the Instrument for Assessing Levels of Physical Activity and Fitness (ALPHA) Environmental Questionnaire [39]. Similar to the process used by a number of other authors [38, 40, 41] the items included in the questionnaire were selected based on their applicability to cycling for recreation and transport. Two items were used to measure *perceived cycling infrastructure*: “there are special lanes, routes or paths for cycling in my neighbourhood”; and “there are cycling routes in my neighbourhood that are separated from traffic”. *Perceived maintenance of bicycle infrastructure* was measured with the one item: “the cycle paths in my neighbourhood are well maintained”. Three items were used to measure *perceived neighbourhood pleasantness and aesthetic*: “my local neighbourhood is a pleasant environment for cycling”; “there is litter or graffiti in the streets of my neighbourhood” (reverse scored); and “in my neighbourhood there are badly maintained, unoccupied or ugly buildings” (reverse scored). Finally, three items were used to measure *perceived network and connectivity*: “cycling is quicker than driving in my neighbourhood during the day”; “there are many road junctions in my neighbourhood”; and “there are many different routes for cycling from place to place in my neighbourhood so I don’t have to go the same way every time”. Each item was measured on a four-point Likert scale (strongly disagree – strongly agree), and a mean score was used in analyses.

Previous studies using the ALPHA Environmental Questionnaire have shown interclass correlation coefficients for individual items used range from 0.54 to 0.82 indicating moderate to good test-retest reliability [42]. Additionally, as suggested by Van Dyck, Deforche, Cardon and De Bourdeaudhuij [43], the definition of the participant’s neighbourhood was modified to “the area you could cycle to in under 15 minutes” rather than walk in 15 min. This increase in buffer size was necessary to account for the increased mobility of cycling [44].

#### Perceived workplace built environment

Perceived workplace built environment was measured using four items developed by Handy and Xing [45]. Participants were asked how true the following statements are about their workplace: “I have access to a shower within a 5-minute walk of my workplace”; “the streets near my workplace are dangerous for cycling” (reverse scored); “there is good transit service near my workplace”; and “it is easy to find a secure rack/post to lock my bike at work”. Each of these items were measured on a four-point Likert scale (not at all true – entirely true), and a mean score was calculated for the analyses.

#### Objective residential density, street connectivity and land-use mix

An objective assessment of land-use mix, residential density and street connectivity was determined using Walk Score™ (referred to as walk score heron). The walk score measures access to amenities within walking distance, population density, block length, and intersection density for all points in a city to give a combined total score between 0 and 100 with a higher score indicating greater walkability [46]. Walk score provides a ranking for each suburb by calculating the walk score for latitudinal and longitudinal grid points approximately 150 m apart across an entire suburb, and providing an average walk score, weighted by population density, for the suburb [46]. Walk score has been validated previously, exhibiting strong and significant correlations with objectively measured street connectivity, residential density, and land-use mix [47, 48].

The suburb ranking for the suburb in which participants lived was used as the objective measure of land-use mix, residential density and street connectivity for cycling for recreation. Since both the home and work neighbourhood environments are associated with active transport [49], a composite ranking of the suburb of which participants lived and the suburb in which they worked was used as the objective measure of the land-use mix, residential density and street connectivity for cycling for transport.

#### Perceived descriptive norms

Perceived descriptive norms were measured using three items [50]. Participants were asked to what extent they agree that the following three referent groups cycle: their closest friends, their family/partner, and their work colleagues. Each of these items were measured on a five-point Likert scale (strongly disagree – strongly agree), and a mean score was calculated for analysis.

#### Perceived injunctive norms

Perceived injunctive norms were measured using three items [50]. Participants were asked to what extent they agree that the following three referent groups accept them cycling: their closest friends, their family/partner, and their work colleagues. Each of these items were answered on a five-point Likert (strongly disagree – strongly agree), and a mean score was calculated for analysis.

#### Perceived group norm

It has been found that descriptive norms are more influential on health behaviours when coupled with an injunctive message [18, 51], so a group norm for each participant was calculated. Consistent with other authors, group norms were operationalized as a combination of descriptive and injunctive norms [33, 52]. Therefore, perceived group norm was calculated by summing the mean score of participant’s perceived injunctive norm and descriptive norm.

### Data analysis

Overall, 3% of the 228 completed cases had some missing data, with the level of missing data for these cases ranging from 3 to 7%. As suggested by Tabachnick and Fidell [53] the expectation maximization method [54] was used to impute missing data. Descriptive statistics were calculated, presenting the mean and standard deviation of scores for each variable for the study population.

Multiple binary logistic regression analyses were conducted to explore the interactive effect of the built environment and social norms on the cycling participation. Each model included cycling for recreation or cycling for transport as the dependent variable and a single built environment variable, a single social norm variable, and the product term of those two variables as the independent variables. All statistically significant odds ratio for product terms could be interpreted as the multiplicative factor of the built environment variable given a 1-standard deviation increase in the social norm variable [55]. To account for the lower power of interactions and, similar to previous studies [27, 28], all interactive effects will be considered significant at *p* < 0.10 and plotted using the excel spreadsheet which automates the steps in plotting interaction effects in logistic regression [56]. Further, because of the exploratory nature of the study, no adjustments were made for multiple hypothesis testing.

Prior to running the models, as suggested by Menard [57] to make each of the models easier to interpret, social norm and built environment variables were standardized to have a mean of 0 and a standard deviation of 1. Each model was adjusted for socio-demographic variables that had a significant association with the outcome variable. Where transport cycling was the outcome, models were adjusted for gender, number of cars in participant’s household, whether participant had regular access to a bicycle, and distance lived from workplace. Models with recreational cycling as the outcome were adjusted for whether participant had regular access to a bicycle.

All data analysis was conducted using SPSS version 24.

## Results

### Descriptive statistics

In total, 83.3% of the sample had completed a bachelor degree or higher, 77.6% lived more than 6kms from their workplace, 86% had regular access to a bicycle, and on average participants had 1.34 (SD = 0.85) cars in their household. Additionally, 53.9% of participants cycled for transport in the last week and 25.4% cycled for recreation in the last week. Descriptive statistics for each of the independent variables for the sample are presented in Table 1.

**Table 1:** Independent variables descriptive statistics

Independent Variable	Mean (SD)
Perceived Neighbourhood Cycling Infrastructure^a^	3.20 (0.73)
Perceived Maintenance of Neighbourhood Cycling Infrastructure^a^	3.08 (0.73)
Perceived Neighbourhood Pleasantness and Aesthetic^a^	2.80 (0.67)
Perceived Cycling Network and connectivity^a^	2.91 (0.56)
Perceived Workplace Built Environment^a^	3.33 (0.47)
Home Suburb Walk Score^b^	74.96 (13.90)
Work Suburb Walk Score^b^	87.97 (8.30)
Descriptive Norm^c^	3.65 (0.79)
Group Norm^d^	8.12 (1.19)

^a^Composite score of items measured on a 4-point Likert scale. Higher score indicates more positive perceptions

^b^A composite ranking from 0 to 100 based on neighbourhood’s land-use mix, residential density and street connectivity. Higher score indicates more walkable neighbourhood

^c^Composite score of items measured in 5-point Likert scale. Higher score indicates more positive norms

^d^Addition of two composite scores of items measured on 5-point Likert scale. Higher score indicates more positive norms

### Built environment and social norm interactions

**Fig. 1:**
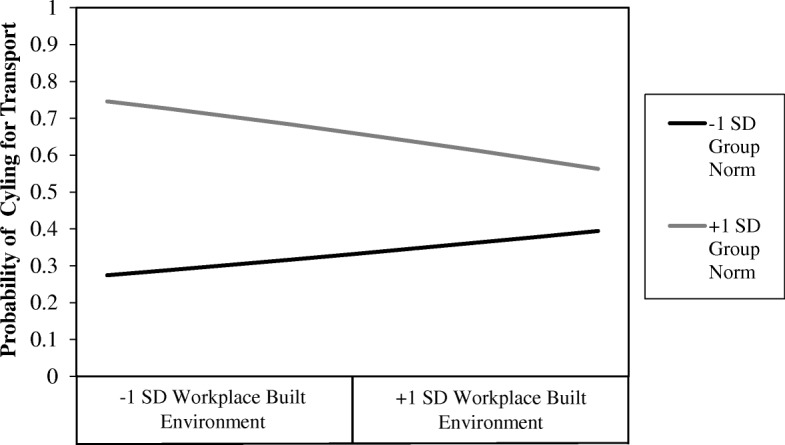
Interactive effect of perceived workplace built environment and perceived group norm on probability of cycling for transport

Twelve models were run to examine the interactive effects of the built environment and perceived social norms with transport cycling as the outcome (Table 2). The interaction between perceived group norm and perceived workplace built environment was significant (*B* = 0.71, *95% CI* = 0.50–1.02, *p* = 0.06). The nature of this interaction indicated that positive perceptions of the workplace built environment can somewhat mitigate the negative influence of unfavourable group norm perceptions. A line graph plotted at +1SD and −1SD group norms illustrates this interaction (Fig. 1).

**Table 2:** Associations of built environment, social norm, and interactions with participation in transport cycling^a^

	Descriptive Norm (DN)		Group Norm (GN)	
		*B [95% CI] p-value*		*B [95% CI] p-value*
Infrastructure (I)	I	0.95 [0.72, 1.32] .863	I	0.98 [0.73, 1.33] .919
	DN	1.82 [1.31, 2.53] < .001	GN	1.93 [1.37, 2.57] < .001
	I*DN	0.98 [0.72, 1.34] .918	I*GN	0.92 [0.64, 1.30] .622
Maintenance (M)	M	1.09 [0.80, 1.48] .593	M	1.09 [0.80, 1.47] .604
	DN	1.82 [1.30, 2.55] < .001	GN	1.91 [1.35, 2.69] < .001
	M*DN	0.83 [0.60, 1.15] .252	M*GN	0.81 [0.58, 1.13] .206
Pleasantness and Aesthetic (PA)	PA	1.12 [0.82, 1.51] .480	PA	1.14 [0.84, 1.54] .412
	DN	1.81 [1.31, 2.52] < .001	GN	1.91 [1.37, 2.67] < .001
	PA*DN	1.03 [0.73, 1.44] .878	PA*GN	0.89 [0.62, 1.29] .539
Network and Connectivity (NC)	NC	1.59 [1.11, 2.26] .011	NC	1.55 [1.08, 2.21] .017
	DN	1.68 [1.20, 2.35] .003	GN	1.55 [1.23, 2.43] .002
	NC*DN	0.99 [0.70, 1.40] .960	NC*GN	0.96 [0.68, 1.36] .825
Workplace Environment (W)	W	0.97 [0.71, 1.33] .864	W	0.93 [0.68, 1.29] .672
	DN	1.81 [1.32, 2.57] < .001	GN	1.98 [1.41, 2.78] < .001
	W*DN	0.76 [0.55, 1.06] .102	W*GN	0.71 [0.50, 1.02] .060
Walk Score (WS)	WS	1.18 [0.79, 1.76] .413	WS	1.08 [0.73, 1.60] .697
	DN	1.82 [1.30, 2.55] < .001	GN	1.88 [1.34, 2.64] < .001
	WS*DN	0.78 [0.51, 1.10] .157	WS*GN	1.00 [0.73, 1.37] .997

^a^All models adjusted for gender, number of cars in participant’s household, whether participant had regular access to a bicycle, and distance lived from workplace

Ten models were run to examine the interactive effects of the built environment and perceived social norms with recreation cycling as the outcome. None of the interactions tested were significant (Table 3).

**Table 3:** Association of built environment, social norms and interactions with participation in recreational cycling^a^

	Descriptive Norm (DN)		Group Norm (GN)	
		*B [95% CI] p-value*		*B [95% CI] p-value*
Infrastructure (I)	I	0.82 [0.62, 1.19] .358	I	0.88 [0.64, 1.20] .415
	DN	1.76 [1.22, 2.56] .003	GN	1.48 [1.04, 2.12] .031
	I*DN	0.87 [0.60, 1.25] .442	I*GN	0.80 [0.55, 1.17] .246
Maintenance (M)	M	0.94 [0.68, 1.29] .684	M	0.95 [0.70, 1.30] .760
	DN	1.76 [1.21, 2.55] .003	GN	1.44 [1.01, 2.04] .043
	M*DN	0.99 [0.71, 1.39] .951	M*GN	0.99 [0.71, 1.37] .936
Pleasantness and Aesthetic (PA)	PA	1.10 [0.79, 1.52] .580	PA	1.13 [0.82, 1.55] .165
	DN	1.74 [1.20, 2.52] .003	GN	1.43 [1.01, 2.04] .044
	PA*DN	1.14 [0.79, 1.64] .495	PA*GN	1.06 [0.72, 1.45] .783
Network and Connectivity (NC)	NC	1.49 [1.03, 2.16] .036	NC	1.46 [1.02, 2.07] .037
	DN	1.73 [1.17, 2.55] .006	GN	1.35 [0.94, 1.94] .106
	NC*DN	0.76 [0.52, 1.13] .173	NC*GN	0.82 [0.57, 1.18] .286
Walk Score (WS)	WS	0.92 [0.66, 1.28] .615	WS	0.90 [0.65, 1.23] .495
	DN	1.76 [1.22, 2.55] .003	GN	1.48 [1.03, 2.11] .033
	WS*DN	0.99 [0.68, 1.43] .943	WS*GN	1.12 [0.82, 1.53] .465

^a^All models adjusted for whether participant had regular access to a bicycle

## Discussion

This study was the first, to the authors’ knowledge, to examine the interactive effects of the built environment and perceived social norms on adult’s participation in cycling for recreation and transport. Results indicated that there was a significant (*p* = 0.06) interactive effect between perceived workplace built environment and perceived group norm on transport cycling. There were no significant interactive effects between the neighborhood built environment and social norms on transport or recreational cycling. These findings provide limited support to the proposition of ecological models that multiple levels of influence interact to influence physical activity behaviours [13, 15].

The positive influence of cycling facilities at the workplace such as showers, changing facilities and secure bike parking on cycling for transport have been reported previously [58, 59]. The interactive effect found in this study suggests that the association between the workplace built environment and transport cycling is stronger among adults with less supportive group norms. Therefore, workplace cycling facilities can somewhat mitigate the negative effect of an unsupportive group norm towards cycling and decrease the influence of group norms on individual’s decision to cycle for transport.

The findings from this study showed that there was no significant interactive effect between perceived and objective measures of the neighbourhood built environment and social norms on transport cycling. These findings are similar to a two previous studies that found no significant interactive effect between walkability and social norms on transport walking behaviours in adults [23], or active transport behaviours of children [27]. A possible explanation for lack of interactive effects on cycling for transport in this study is because, on average, participants in this study lived in very walkable neighbourhoods. This means that destinations in many participant’s neighbourhoods were easily accessible by walking, which may make cycling to destinations in their neighbourhood superfluous, if not inconvenient [60]. In addition to living in very walkable neighbourhoods, the vast majority of the participants travelled into the Central Business District or inner suburbs of Melbourne for work, meaning that there was a notable lack of variability in attributes of the built environment between participants. Also, over three-quarters of participants reported living more than 6kms from their workplace. Therefore, it is possible that a large portion of the area that participants cycled for transport was between the neighbourhood they live and work, which was not captured in this study.

This study also showed that there were no significant interactive effects between perceived or objective measures of the neighbourhood built environment and perceived social norms on recreational cycling. This finding is akin to the findings of a study on children that found no significant interaction between social norms from participant’s parents and walkability on cycling in leisure time [27]. Similarly, another study found that there was no interactive effect between social norms and walkability on walking for recreation in adults [23]. Interestingly, a number of studies have found significant interactive effects between the built environment and social support. Two studies reported and interactive effect that shows that a supportive built environment can somewhat negate the negative effects of a lack of social support on recreational physical activity [19, 22]. Other studies found an interaction between social support and the neighbourhood built environment whereby social support and the built environment reinforced each other to encourage recreational physical activity [20, 27]. These findings further demonstrate that social norms and social support interact differently with the built environment to influence recreational physical activity. The difference in findings between studies supports the proposition of ecological models, that the influence of variables are very behavioural and context specific [29].

### Strengths and limitations

A major strength of the current study was the use of validated and conceptually relevant measures of the built environment and cycling participation. Another strength of this study was that a composite ranking of the home and work neighbourhood built environment were included in transport cycling models. Additionally, both subjective and objective measures of the built environment were used. However, the current study also had some limitations that should be considered when interpreting the results. First, despite being validated, walk score remains a novel method for measuring the built environment and does not measure cycling specific components of the built environment. Additionally, measures used in this study did not capture the objective built environment between the neighbourhoods that the participants lived and worked, which may have influenced participant’s decision to cycle for transport. This could partly explain the absence of interactive effects between the objective built environments and social norms in this study. Second, the cross-sectional study design means that conclusions from this study can only infer association rather than causation. Third, this study relied upon self-reported measures of cycling, which may be influenced by recall bias and social desirability [61]. Fourth, there were some limitations associated with the sample. Although the sample size was appropriate for the type of data analysis conducted [62], the relatively small sample size may have increased the likelihood of type II errors. Also, participants in this study were recruited from a convenience sample, which may beget selection bias, evident by cycling rates in this study being greater than the national average for Australia, and limit the generalisability of the results. Finally, this was an exploratory study so no adjustments were made for multiple hypothesis tests.

## Conclusion

The present study provides limited support for the interactive effects between the built environment and social norms on cycling participation. Only the interactive effect between the workplace built environment and group norm was significant. The nature of this interaction suggests that positive perceptions of the workplace built environments may somewhat compensate for the effects of negative group norm perceptions. Given the potential health enhancing benefits of increased cycling participation, further research into how other socio-ecological factors interact to influence cycling participation warrants further attention. Findings could guide the development of interventions to increase cycling participation in the future.

## Additional file

Additional file 1: Online questionnaire. (DOCX 19 kb)

## Abbreviations

ALPHA: Assessing Levels of Physical Activity and Fitness Questionnaire
IPAQ-LF: International Physical Activity Questionnaire Long Form
