# Supplementary material for: An exploratory analysis of the interactions between social norms and the built environment on cycling for recreation and transport
Source: BMC Public Health. 2018 Oct 5;18:1162. doi: 10.1186/s12889-018-6075-4 (PMC6173852; doi:10.1186/s12889-018-6075-4)
Supplement: Supplementary file 1 — Online questionnaire. (DOCX 19 kb) [file 12889_2018_6075_MOESM1_ESM.docx]

| 1.1 What is your gender? | - Female - Male - My gender is not listed - I wish not to answer |
| --- | --- |
| 1.2 What is your date of birth | dd/mm/yyyy |
| 1.3 Education – What is the highest level of education you have completed? | - I did not complete secondary school - Senior Secondary School Certificate - Certificate Degree - Bachelor Degree or Higher |
| 1.4 How many cars do you have in your household? | Type in number |
| 1.5 Do you have regular access to a bicycle? | - Yes - No |
| 1.6 How far do you live from your workplace? | - <1 km - 1-5km - 6-10km - >10km |
| 1.7 What suburb do you live in? | Type in suburb |

**Online Questionnaire**

**Section 1 – Socio-demographic questions**

**Section 2 – Cycling behaviour questions**

2.1 This question is about how you travelled from place to place, including to places **like work,**

**shops, public transport**, and so on.

During **the last 7 days**, on how many days did you bicycle for at least 10 minutes at a time to go from place to place? This **does not** include time spent cycling for exercise or recreation.

_____ days

How much time did you usually spend on one of these days to bicycle from place to place?

_____ minutes per day

2.2 This question is about all the bicycling that you did in the last 7 days solely for

**recreation, sport, exercise or leisure**. Please do not include any activities you have already

mentioned.

During **the last 7 days**, on how many days did bicycle for at least 10 minutes in your leisure time.

_____ days

How much time did you usually spend on one of these days bicycling for recreation, sport, exercise, or leisure?

_____ minutes per day

**Section 3 – Neighbourhood perceived environment questions**

3. How true are the following statement in describing your neighbourhood?

|  | Strongly disagree | Disagree | Agree | Strongly agree |
| --- | --- | --- | --- | --- |
| 3.1 There are special lanes, routes or paths for cycling in my neighbourhood | - 1 | - 2 | - 3 | - 4 |
| 3.2 There are cycling routes in my neighbourhood that are separated from traffic | - 1 | - 2 | - 3 | - 4 |
| 3.3 The cycle paths in my neighbourhood are well maintained | - 1 | - 2 | - 3 | - 4 |
| 3.4 My local neighbourhood is a pleasant environment for cycling | - 1 | - 2 | - 3 | - 4 |
| 3.5 There is litter or graffiti in the streets of my neighbourhood | - 1 | - 2 | - 3 | - 4 |
| 3.6 In my neighbourhood there are badly maintained, unoccupied or ugly buildings | - 1 | - 2 | - 3 | - 4 |
| 3.7 Cycling is quicker than driving in my neighbourhood during the day | - 1 | - 2 | - 3 | - 4 |
| 3.8 There are many road junctions in my neighbourhood | - 1 | - 2 | - 3 | - 4 |
| 3.9 There are many different routes for cycling from place to place in my neighbourhood so I don’t have to go the same way every time | - 1 | - 2 | - 3 | - 4 |

**Section 4 - Workplace built environment questions**

|  | Not at all true | Somewhat true | Mostly true | Entirely true |
| --- | --- | --- | --- | --- |
| 4.1 I have access to a shower within a 5-minute walk of my workplace | - 1 | - 2 | - 3 | - 4 |
| 4.2 The streets near my workplace are dangerous for cycling | - 1 | - 2 | - 3 | - 4 |
| 4.3 There is good transit service near my workplace | - 1 | - 2 | - 3 | - 4 |
| 4.4 It is easy to find a secure rack/post to lock my bike at work | - 1 | - 2 | - 3 | - 4 |

**Section 5 – Perceived descriptive norms questions**

5. To what extent do you agree that each of the following groups cycle.

|  | Strongly Disagree | Disagree | Neutral | Agree | Strongly agree |
| --- | --- | --- | --- | --- | --- |
| 5.1 Family/ Partner | - 1 | - 2 | - 3 | - 4 | - 5 |
| 5.2 Friends | - 1 | - 2 | - 3 | - 4 | - 5 |
| 5.3 Work Colleagues | - 1 | - 2 | - 3 | - 4 | - 5 |

**Section 6 -Perceived injunctive norms questions**

6. To what extent do you agree each of the following groups approve of you cycling?

|  | Strongly disagree | Disagree | Neutral | Agree | Strongly agree |
| --- | --- | --- | --- | --- | --- |
| 6.1 Family/ Partner | - 1 | - 2 | - 3 | - 4 | - 5 |
| 6.2 Friends | - 1 | - 2 | - 3 | - 4 | - 5 |
| 6.3 Work Colleagues | - 1 | - 2 | - 3 | - 4 | - 5 |
